# Protease Activity Increases in Plasma, Peritoneal Fluid, and Vital Organs after Hemorrhagic Shock in Rats

**DOI:** 10.1371/journal.pone.0032672

**Published:** 2012-03-27

**Authors:** Angelina E. Altshuler, Alexander H. Penn, Jessica A. Yang, Ga-Ram Kim, Geert W. Schmid-Schönbein

**Affiliations:** Department of Bioengineering, The Institute of Engineering in Medicine, University of California, San Diego, La Jolla, California, United States of America; Duke University Medical Center, United States of America

## Abstract

Hemorrhagic shock (HS) is associated with high mortality. A severe decrease in blood pressure causes the intestine, a major site of digestive enzymes, to become permeable – possibly releasing those enzymes into the circulation and peritoneal space, where they may in turn activate other enzymes, e.g. matrix metalloproteinases (MMPs). If uncontrolled, these enzymes may result in pathophysiologic cleavage of receptors or plasma proteins. Our first objective was to determine, in compartments outside of the intestine (plasma, peritoneal fluid, brain, heart, liver, and lung) protease activities and select protease concentrations after hemorrhagic shock (2 hours ischemia, 2 hours reperfusion). Our second objective was to determine whether inhibition of proteases in the intestinal lumen with a serine protease inhibitor (ANGD), a process that improves survival after shock in rats, reduces the protease activities distant from the intestine. To determine the protease activity, plasma and peritoneal fluid were incubated with small peptide substrates for trypsin-, chymotrypsin-, and elastase-like activities or with casein, a substrate cleaved by multiple proteases. Gelatinase activities were determined by gelatin gel zymography and a specific MMP-9 substrate. Immunoblotting was used to confirm elevated pancreatic trypsin in plasma, peritoneal fluid, and lung and MMP-9 concentrations in all samples after hemorrhagic shock. Caseinolytic, trypsin-, chymotrypsin-, elastase-like, and MMP-9 activities were all significantly (p<0.05) upregulated after hemorrhagic shock regardless of enteral pretreatment with ANGD. Pancreatic trypsin was detected by immunoblot in the plasma, peritoneal space, and lungs after hemorrhagic shock. MMP-9 concentrations and activities were significantly upregulated after hemorrhagic shock in plasma, peritoneal fluid, heart, liver, and lung. These results indicate that protease activities, including that of trypsin, increase in sites distant from the intestine after hemorrhagic shock. Proteases, including pancreatic proteases, may be shock mediators and potential targets for therapy in shock.

## Introduction

Trauma is associated with high mortality [1]. One major cause of death in trauma patients is hemorrhagic shock (HS) [2], during which the intestine is underperfused [3]. As a result of ischemia, intestinal permeability increases [4], allowing luminal content including pancreatic digestive enzymes, to escape from the lumen into the wall of the intestine [5], [6]. Proteases that have penetrated the barrier can further increase the overall proteolytic activity in the intestinal wall by activating MMPs [6], [7]. Pretreatment in the intestinal lumen with a broad spectrum serine protease and lipase inhibitor (nafamostat mesilate, ANGD), reduces circulating neutrophil activation [8] and delays or prevents mortality in experimental shock models indicating the intestine as a key organ to preserve during shock [9]–[10]. One of the possible effects of ANGD in the lumen of the intestine could be to prevent active pancreatic proteases from entering the circulation, by helping to preserve the mucosal barrier and/or by inhibiting proteases that enter the intestinal wall.

Shock mediators entering or formed in the wall of the intestine may be transported out of the intestine via the portal venous system, the intestinal lymph, or by passive transport through the intestinal wall into the peritoneum [11], [12]. It is unknown whether digestive enzymes are among the mediators transported out of the intestine into the systemic circulation and other organs during hemorrhagic shock. Should this occur, uncontrolled proteolytic activity in compartments outside the lumen of the intestine could lead to cleavage of important plasma proteins and/or cell surface receptors contributing to the morbidity and possible mortality of the animal [13]. In pancreatitis, which has similarities to shock and may also result in multi-organ failure, plasma trypsin levels have been correlated with mortality [14]. In shock, pancreatic amylase and lipase have been measured in plasma and predict mortality [15], but the presence and activity of pancreatic *proteases* remains to be determined.

It has been hypothesized that, if released into the systemic circulation, pancreatic proteases will not be active due to binding to plasma protease inhibitors (serpins; e.g. α_2_-macroglobulin, α_1_-antitrypsin, etc.) [16]. However, the blocking ability of serpins is limited. They may be saturated, and it has been shown that while binding to these inhibitors prevents proteases like trypsin from digesting large proteins, smaller peptides are still cleavable [17]. Thus, proteins with exposed loops or terminal ends may still be at risk for proteolytic degradation [13].

Because of possible binding to vascular antiproteases, measurements of the potential for proteolysis in shock plasma may be negatively skewed if the selected test substrate is a large globular protein such as casein, a substrate commonly used to measure non-specific protease activity. Additionally, we have observed that serine protease activity in plasma samples will decrease with increasing storage time (−20°C) and/or freeze-thaw cycles, further increasing the difficulty of obtaining a complete spectrum of the proteolytic potential in shock plasma [18]. Therefore, we recognized a need to measure small peptide proteolytic activity on fresh samples after hemorrhagic shock.

The objectives of this study were to determine in experimental hemorrhagic shock (1) select protease activities and concentrations outside of the intestine (plasma, peritoneal fluid, vital organs) by plate zymography, gel zymography, and immunoblotting and (2) whether the mechanism by which serine protease inhibition in the intestinal lumen provides protection in hemorrhagic shock is due to reduction of protease activity in the systemic circulation. Our results indicate that protease activity and levels are elevated after hemorrhagic shock. While ANGD treatment reduces neutrophil infiltration into organs distant from the intestine, it does not reduce protease activity in the tissues tested at 2 hours after hypotension.

## Methods

### Animal Protocol and Tissue Collection

The animal protocols were reviewed and approved by the University of California, San Diego Animal Subjects committee (Protocol Number S01113). Male Wistar rats (body weight between 270 and 460 g; 347±49 g [mean±sd], Harlan, Indianapolis, IN) were administered general anesthesia (xylazine, 20 mg/kg i.m., followed 20 minutes later by sodium pentobarbital, 50 mg/kg i.m.). After laparotomy, 2 ml saline were injected into the peritoneal space then drained after 2 minutes. The lavage fluid was collected in a tube containing 20 µl heparin and centrifuged (1000 g, 5 min) to remove any accumulated cells. Next, the left femoral vein and artery were cannulated. We then injected either saline (*HS+SAL* group) or a serine protease inhibitor (6-amidino-2-naphtyl *p*-guanidinobenzoate dimethanesulfate, ANGD, *nafamostat mesilate*, Torii Pharmaceutical, Chiba, Japan; *HS+ANGD* group; 2 mg/ml ANGD in 100 mg/ml D-glucose in saline) into the lumen of the small intestine (7–10 ml at two to three injection sites to fill the small intestine but avoid circumferential stretching). To prevent clotting in catheters and shed blood, animals were heparinized (10 U/ml i.v., assuming 6% blood volume per body weight) prior to inducing hemorrhagic shock.

Mean arterial blood pressure (MAP) was reduced to 35 mmHg by withdrawing blood through the venous catheter (0.2 ml/min). This pressure was maintained by further withdrawal/return of blood over a 2-hour ischemic period. The first two milliliters of shed blood were collected, centrifuged (1000 g, 5 min), and stored at 4°C as pre-hemorrhagic shock (*Pre-HS*) plasma. After 2 hours, the remaining shed blood plus 2 ml of heparinized saline (to replace the 2 ml of pre-ischemic blood) was reinfused (0.5 ml/min i.v.), and the animal observed for 2 hours. At the end of this reperfusion period, 4 ml of post-hemorrhagic shock (*Post-HS*) blood was collected, centrifuged (1000 g, 5 min) and plasma aliquots were either immediately measured for protease activity or frozen (−20°C). An aliquot of post-HS plasma received an extra 10 U/ml heparin before centrifugation. Post-peritoneal lavage fluid was collected in the same manner as described above. The brain, heart, liver, and lung were collected immediately after euthanasia (120 mg/kg sodium pentobarbital i.v.) and snap frozen for later protease assays, gelatin zymography, and immunoblot analysis. Separate animals (*No-HS* group) were euthanized immediately after femoral catheterization for control brain, heart, liver, and lung tissue samples. Given the limited availability of Pre-HS plasma, some assays were performed using remaining unpaired Pre-HS plasma (i.e. Pre-HS plasma drawn from animals in both the HS+SAL and HS+ANGD groups).

Brain, heart, liver, and lung were homogenized (0.1 g tissue/ml) in phosphate buffered saline (PBS) (pH 6) containing 0.5% hexadecyltrimethyl bromide (HTAB) for myeloperoxidase (MPO) assays, MMP-1/9 assays, and gelatin gel zymography. Tissues were homogenized for immunoblot analysis in CelLytic buffer (Sigma-Aldrich, St. Louis, MO) with protease inhibitor cocktail (Sigma-Aldrich). Homogenates were centrifuged (1.4×10^4^ g, 4°C, 30 min) and the supernatants collected, aliquoted, and stored at −80°C. After removal from −80°C, protein concentrations were measured (BCA protein assay kit, ThermoScientific, Waltham, MA) prior to use of samples in subsequent experiments.

### Microplate Assays

All microplate assays were performed in triplicate in 96 well black-sided flat bottom polystyrene plates (Corning, New York, NY) using a microplate reader (FilterMax F-5 Multi-mode, Molecular Devices, Sunnyvale, CA).

Since shed blood was held outside the body for 2 hours during the ischemic period, we performed a pilot study to confirm that any increased protease activity after reperfusion was not due to increases *in-vitro* in the shed whole blood. We compared freshly drawn blood to blood that had been stored for 2 hours for all protease activity measurements. No differences were detected.

#### Myeloperoxidase (MPO) Activity Assay

As a measure of the degree of inflammation after shock, we measured myeloperoxidase activity for neutrophil infiltration/accumulation in lung, liver, heart, and brain. 20 µl of 1 mg/ml brain, heart, liver, or lung homogenate, were added to 180 µl of 0.167 mg/ml o-dianisidine dihydrochloride (Sigma-Aldrich) and 0.005% H_2_O_2_ in PBS (pH 6). We measured absorbance at 450 nm every 5 minutes for 1 hour at 37°C. MPO activity is presented as the change in absorbance per minute per milligram of protein.

#### Trypsin-, Chymotrypsin-, and Elastase-like Activity Measurements

The ability of shock plasma and peritoneal lavage fluid to digest small peptides was determined using benzoylarginyl-p-nitroanilide hydrochloride (BAPNA, Sigma-Aldrich), N-succinyl-L-phenylalanine-p-nitroanilide (SPNA, Sigma-Aldrich), N-succinyl-alanine-alanine-proline-leucine-p-nitroanilide;L-leucinamide, N-(3-carboxy-1-oxopropyl)-L-alanyl-L-alanyl-L-prolyl-N-(4-nitrophenyl) (SAAPLPNA, Sigma-Aldrich), which are substrates for trypsin-like, chymotrypsin-like, and elastase-like enzyme activity, respectively. Stock solutions were prepared in dimethylsulfoxide (DMSO) (BAPNA-20 mM, SPNA-100 mM, and SAAPLPNA-84.7 mM). Stock solutions were then diluted in PBS to their working solution concentrations (0.5 mM for BAPNA, 5 mM for SPNA, and 0.5 mM for SAAPLPNA). 20 µl of fresh plasma, peritoneal fluid, or PBS as control were added to either 180 µl of PBS (containing the same percentage of DMSO as substrates) for control or working solution of BAPNA, SPNA, or SAAPLPNA, respectively. Light absorbance readings (at 405 nm) were made at time = 0 hr and 24 hrs (resulting measurements were well below the maximum absorbance for each substrate). Post hemorrhagic shock plasma samples were susceptible to light diffraction due to colloid material (opacity) if allowed to evaporate. Therefore, plates were kept covered at room temperature. Opacity was also minimized if post shock blood had been treated with additional heparin, suggesting clotting was a reason for the opacity in the samples. A normalized absorbance was computed as: A_n_ = ΔA_Sa+Sub_−ΔA_Sa_−ΔA_Sub_ where ΔA_Sa+Sub_ is the change in absorbance of the sample+substrate, ΔA_Sa_ is the change in absorbance of the sample+PBS w/ DMSO, and ΔA_Sub_ is the change in absorbance of the substrate+PBS.

To convert trypsin-, chymotrypsin-, and elastase-like activities into equivalent uninhibited concentrations of each enzyme, purified bovine trypsin (Sigma-Aldrich), chymotrypsin (Sigma-Aldrich), and elastase (Worthington Biochemical Corporation, Lakewood, NJ) were serially diluted (micromolar to femtomolar range) and added to their respective substrates. The plates were read at 24 hours and a calibration curve generated to estimate the equivalent uninhibited concentration, based on proteolytic activity, in the samples. 24 hours of incubation was necessary to achieve adequate signal to noise ratios with these substrates even when using pure enzymes.

#### Casein Protease Activity Assay

To assess the ability of proteases in plasma and peritoneal lavage fluid to digest large globular proteins, we utilized the Enzchek protease assay kit (Invitrogen, Carlsbad, CA), which consists of casein internally quenched with Texas Red fluorophores (Ex/Em: 589/617 nm) reconstituted in a digestion buffer. One part fresh plasma or peritoneal fluid was added to four parts digestion buffer and five parts casein substrate (F_Sample+Casein_). To determine how much of the measured activity was due to serine proteases, a second aliquot of each sample received 1 mM (final concentration) phenylmethylsulfonyl fluoride (PMSF), a serine protease inhibitor. To correct for absorption or auto-fluorescence of the sample at 617 nm, we mixed a known quantity of Texas Red (Vector Laboratories, Burlingame, CA) in digestion buffer 9∶1 with sample (F_Tx+Sample_) or digestion buffer (F_Tx+Buffer_). Samples were incubated for 100 min at 37°C in a microcentrifuge tube before pipetting the reaction mixture to the microplate and reading fluorescence. Caseinolytic activity (Relative Fluorescent Units; RFU) was determined as: (F_Tx+Buffer_/F_Tx+Sample_)*(F_Sample+Casein_).

#### MMP-1/9 Activity Measurements

Pilot studies revealed that, unlike other activities we examined, MMP-1/9 activity was not diminished by storage at −20°C, which permitted the use of stored plasma and peritoneal fluid. Plasma (100-fold final dilution) or peritoneal fluid (50-fold final dilution) samples were loaded into a 96 well plate with fluorescent MMP-1/9 substrate (American Peptide Company, Sunnyvale, CA) diluted to a final concentration of 10 µM in digestion buffer (100 mmol NaCl, 23 mmol Tris HCl, 2.4 mmol CaCl_2_, 5 µM ZnCl_2_, 0.01% Brj 35, pH 7.6, with 1 mM PMSF to inhibit any residual serine protease activity). Samples with digestion buffer only (no substrate) served as control for autofluorescence. Sample fluorescence was measured every 5 minutes for 30 minutes (Ex/Em: 365/450 nm). Activity was reported as the initial change of substrate fluorescence per unit time (relative fluorescent units per minute).

### Gelatin Zymography

As a first step towards identification, we employed gelatin gel zymography to determine molecular weights of prominent proteases appearing in tissue homogenates, stored plasma, and stored peritoneal fluid after hemorrhagic shock. It should be noted that gel zymography characteristically separates all proteases from non-covalently bound inhibitors (pilot studies revealed this includes the binding of ANGD to trypsin, chymotrypsin, or elastase) and can also activate some pro-enzymes to become capable of cleaving the gelatin substrate.

For tissue homogenates, 30 µg of protein was added to equal volume of Laemmli sample loading buffer (Bio-Rad, Hercules, CA). Either 0.5 µl of plasma or 2 µl of peritoneal fluid were diluted in 8 µl sample loading buffer before loading the gel. Samples were separated by gel electrophoresis on a 4% stacking gel and 10% SDS-PAGE resolving gel containing 80 µg/ml porcine gelatin (Sigma-Aldrich) as the protease substrate. After protein separation, proteins in the gels were renatured in four 15-minute washes of 2.5% Triton X-100 solution. Gels were incubated overnight at 37°C in developing buffer (0.05 M Tris base, 0.2 M NaCl, 4 µM ZnCl_2_, 5 mM CaCl_2_·2H_2_O). Following incubation, gels were stained (50% methanol, 10% acetic acid, 40% water, and 0.25% Coomassie blue solution) for three hours. Gels were destained (50% methanol, 10% acetic acid, and 40% water solution) until the first appearance of bands, then transferred to water for rehydration before acquiring images.

To confirm the low molecular weight bands (20–30 kDa) were serine proteases, gels were run in duplicate. One gel was renatured as described above. The second gel was renatured and developed in buffers containing 200 µg/ml of the serine protease inhibitor, ANGD. Pilot studies with pure trypsin, chymotrypsin, or elastase, with and without ANGD, confirmed that ANGD can inhibit each enzyme as measured by plate zymography.

To determine trypsin-specific activity of lower bands, select gels were also renatured and developed in 100 µM tosyl-lysine chloromethyl ketone hydrochloride (TLCK) (Sigma-Aldrich), a specific inhibitor of trypsin-like enzymes.

The gel analysis function in ImageJ (http://rsbweb.nih.gov/ij/) was used to quantify protease activity bands by densitometry. Values are reported in Relative Intensity Units (RIU).

### Immunoblotting

Based on results from our lab suggesting that trypsin is better able to penetrate into the wall of an ischemic intestine than chymotrypsin or elastase [19], and on preliminary results from this study suggesting MMP-9 activity is increased in HS, we focused our immunoblot studies on those two proteases.

To determine the relative concentrations of MMP-9 and pancreatic trypsin in plasma, peritoneal fluid, and tissue homogenates, each fluid was first denatured by mixing 1∶1 with 2× sample loading buffer (Bio-Rad) containing the reducing agent β-mercaptoethanol (0.05% by volume) before boiling for 10 minutes. 2 µl (MMP-9 assays) or 4 µl (trypsin assays) of denatured plasma solution or 10 µl denatured peritoneal fluid solution was loaded per well into an SDS-PAGE gel (8% resolving, 4% stacking for MMP-9; 12% or precast 4–20% gels from Bio-Rad for trypsin). For detection of MMP-9 and trypsin in tissue homogenates we loaded 40 µg and 80 µg of protein per well, respectively. After separation, proteins were transferred onto a nitrocellulose membrane (Bio-Rad). Membranes were blocked with 5% bovine serum albumin in tris-buffered saline with 0.5% Tween-20 (TBS-T). Primary antibodies against MMP-9 (1∶1000, ab-76003, Abcam, Cambridge, MA), pancreatic trypsin (1∶1000, sc-137077, Santa Cruz Biotechnology, Santa Cruz, CA), GAPDH (1∶2000; sc-48167) and β-actin (1∶2000, sc-130301, Santa Cruz Biotechnology) were diluted in 1% BSA in TBS-T and incubated with membranes overnight at 4°C on a rotary shaker (60 cycles/minute). Primary antibodies were washed with TBS-T (3×, 10 min) before application of anti-goat, -rabbit, or -mouse secondary antibodies (1∶3000; Santa Cruz Biotechnology). Secondary antibodies were also washed in TBS-T (3×, 10 min). Bands were detected using enhanced chemiluminescence (ECL) pico substrate (Thermo Scientific) for all proteins except for plasma and lung pancreatic trypsin and brain MMP-9, which were detected using the femto substrate (Thermo Scientific). Bands were quantified in ImageJ by densitometry and expressed as pico Relative Units (RU) or femto RU based on the type of ECL detection utilized.

### Statistical Analysis

Results are presented as mean ± standard deviation (n = 5 or 6 rats per group). We used paired t-tests or Mann-Whitney tests to compare plasma and peritoneal fluid values before versus after hemorrhagic shock (in individual animals) and unpaired t-tests to compare HS+SAL versus HS+ANGD groups. Unpaired t-tests or Mann-Whitney tests were used to compare tissue samples of the No-HS group with HS+SAL or HS+ANGD shock groups or when insufficient Pre-HS plasma remained to perform a paired assay. P<0.05 was considered statistically significant.

## Results

### Inflammation and Intestinal Damage in Hemorrhagic Shock

**Figure 1:**
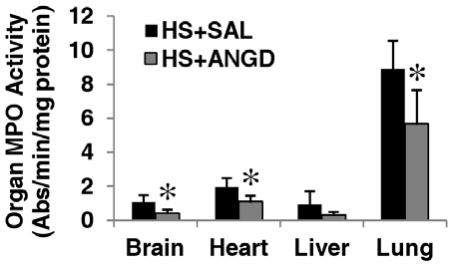
Myeloperoxidase activity. MPO activity in tissue homogenates at 2 hours after hemorrhagic shock. N = 5 rats per group. *p<0.05 by t-test vs. HS+SAL.

ANGD treatment reduced inflammation after shock as measured by MPO activity (i.e. neutrophil infiltration) in brain, heart, and lung tissues, but not significantly in liver homogenates at two hours after reperfusion (P = 0.18) (Fig. 1). ANGD treated animals also had fewer macroscopic lesions on the intestine in the form of visible red cell microhemorrhages into the interstitium after hemorrhagic shock compared to saline treated animals (not shown).

### Protease Activity Detected by Small Peptide Substrates but not Casein Substrate

**Figure 2:**
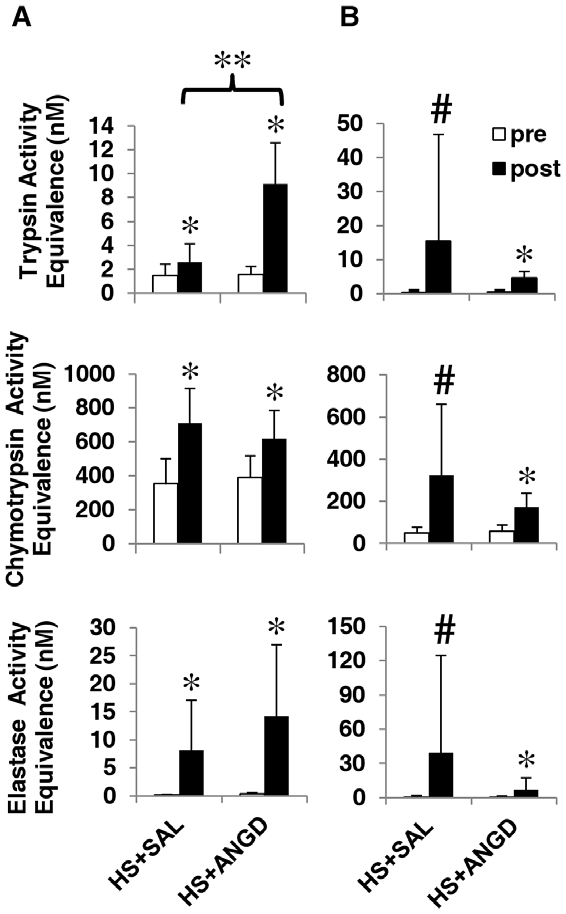
Plasma and peritoneal fluid serine protease activity. Trypsin-, chymotrypsin-, and elastase-like activities in (**A**) plasma and (**B**) peritoneal fluid expressed as equivalent activity to concentrations of pure trypsin, chymotrypsin, and elastase. Note the difference in scales of the ordinate. N = 6 rats per group. *p<0.05, paired t-test comparing pre vs. post HS+SAL or HS+ANGD; **p<0.05 by t-test vs. post HS+SAL; #p<0.05, paired Mann-Whitney test comparing pre vs. post HS+SAL or HS+ANGD.

When using the small peptide substrates BAPNA, SPNA, and SAAPLPNA to assess protease activity, we observed that post-shock plasma trypsin-, chymotrypsin-, and elastase-like activities were all elevated after shock compared to low activity levels in pre-shock controls (Fig. 2A). Trypsin-, chymotrypsin-, and elastase-like activities in peritoneal fluid were also significantly elevated after shock, though variability was enhanced in the HS+SAL group due to individual cases with high protease activity (Fig. 2B). Overall, trypsin and elastase activity levels were higher in the peritoneal fluid compared to plasma, though this elastase activity could be due in part to elastase released from neutrophils. ANGD had no significant effect on peritoneal fluid protease activity or on plasma protease activity other than to increase post-shock trypsin activity.

**Figure 3:**
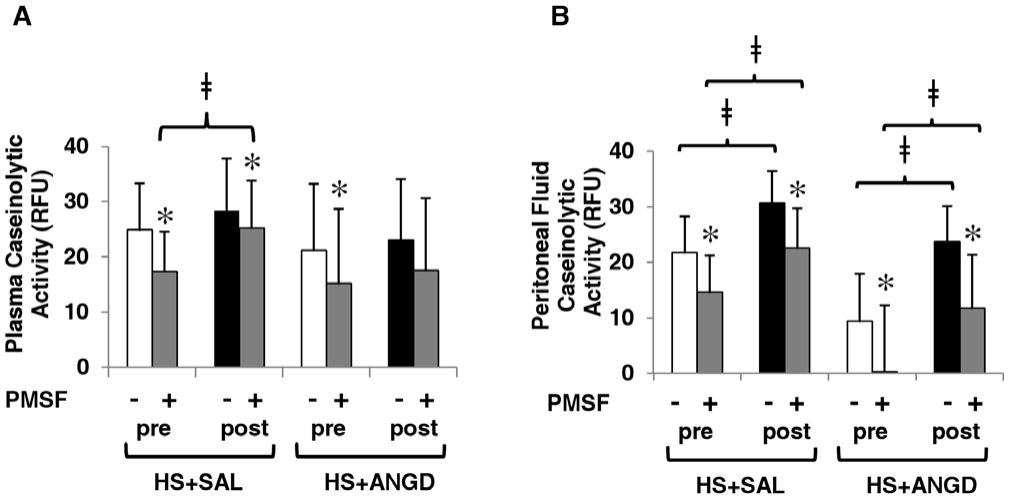
Plasma and peritoneal fluid caseinolytic activity. Caseinolytic activity with and without addition of a serine protease inhibitor, 1 mM PMSF, of (**A**) plasma and (**B**) peritoneal fluid pre/post shock with saline or ANGD pre-treatment in the intestinal lumen. Protease activity was detectable, but low with this substrate. N = 5 rats per group. *p<0.05 by paired t-test vs. without PMSF; ≠p<0.05 by paired t-test comparing pre vs. post plasma or peritoneal fluid from HS+SAL or HS+ANGD animals.

When general protease activity was measured using a casein substrate, we saw no significant difference in plasma protease activity two hours after HS compared to before HS (with or without ANGD) (Fig. 3A). We saw a significant increase in protease activity after shock in peritoneal fluid (Fig. 3B), and the caseinolytic activity in post-shock peritoneal fluid correlated well with MAP at 2 hours post-HS (correlation coefficient = 0.86). ANGD treatment did not significantly reduce protease activity in peritoneal fluid compared to saline-treated animals. Interestingly, PMSF significantly lowered the remaining protease activity in nearly all cases, suggesting a portion was of serine protease origin. There was an increase in PMSF-resistant protease activity over the pre- to post-shock time period in the plasma of the HS+SAL group, but not the plasma of the HS+ANGD group, in which activity levels did not noticeably increase after shock (Fig. 3A).

Given the tendency of post-shock plasma to clot if the small-peptide zymography plates were not covered during digestion (see Methods), we sought to determine whether some of the measured increase in protease activity could be due to activation of proteases in the clotting cascade. Supplemental heparin added to the post-shock blood only affected chymotrypsin-like activity measured with SPNA (from 0.17 to 0.13 normalized absorbance, P<0.04). Similarly, there was a tendency for post-shock plasma (only) to cause a gel to form during the casein digestion. This gel was easily disrupted and did not form if the post-shock blood was supplemented with heparin, which prevented coagulation. Supplemental heparin gave similar results to disrupted gels (not shown).

### Peripheral Trypsin Detection

**Figure 4:**
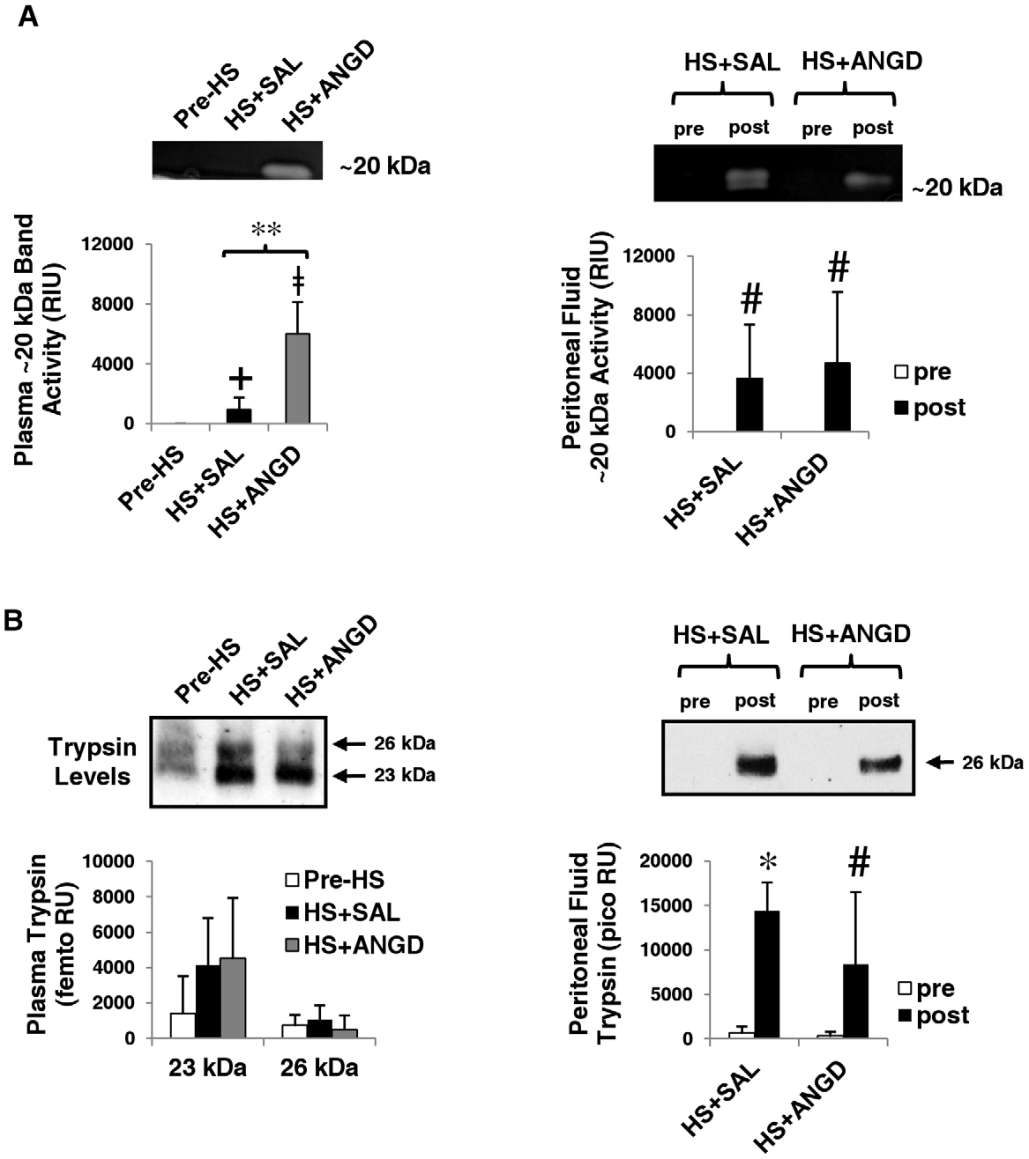
Plasma and peritoneal fluid gel zymography and trypsin levels. (**A**) Gelatin gel zymography detection and quantification in Relative Intensity Units (RIU; bar graph beneath) of ∼20 kDa bands for plasma (right; N = 6, 6, 5 rats per group) and peritoneal fluid (left; N = 5 rats per group). Bands do not form or are greatly reduced when gels are renatured with ANGD or TLCK, respectively (not shown). (**B**) Immunoblot detection and quantification (bar graph beneath) of trypsin in plasma (left) and peritoneal fluid (right), revealing the 26 and 23 kDa isoforms of trypsin that are present. +p<0.05 by Mann-Whitney test vs. Pre-HS; ≠p<0.05 by t-test vs. Pre-HS; **p<0.05 by t-test HS+ANGD vs. HS+SAL; #p<0.05 pre vs. post HS+SAL or HS+ANGD by paired Mann-Whitney test; *p<0.05 by paired t-test comparing pre vs. post HS+SAL.

Low molecular weight bands around 20 kDa (the approximate molecular weight of pancreatic serine proteases) were also detected in the plasma and peritoneal fluid by gelatin gel zymography (Fig. 4A). These low molecular weight bands did not appear when the gels were renatured and developed in buffer containing ANGD, suggesting they could potentially be trypsin, chymotrypsin, and elastase. In further support of this hypothesis, renaturing and developing in buffer containing TLCK, a trypsin-like enzyme inhibitor, also caused a substantial reduction in the appearance of the bands (not shown). Interestingly, ANGD treated animals had significantly higher levels of this band in the plasma suggesting the band corresponds primarily to trypsin activity, given our trypsin results in Fig. 2A.

Two isoforms of pancreatic trypsin were detected in plasma by immunoblot at 23 and 26 kDa. Variability was great for the 23 kDa band, which required femto ECL to detect. We observed a non-significant trend for increased presence of this band after HS (P = 0.07 for HS+SAL and P = 0.11 for HS+ANGD by Mann-Whitney test). No trend was observed for the 26 kDa band in plasma. The 26 kDa isoform of trypsin in the peritoneal fluid was significantly increased in the HS+SAL group and to a lesser, though still significant, extent in the HS+ANGD group (Fig. 4B right; P = 0.15 for HS+SAL vs. HS+ANGD post-shock peritoneal fluid by Mann-Whitney test).

**Figure 5:**
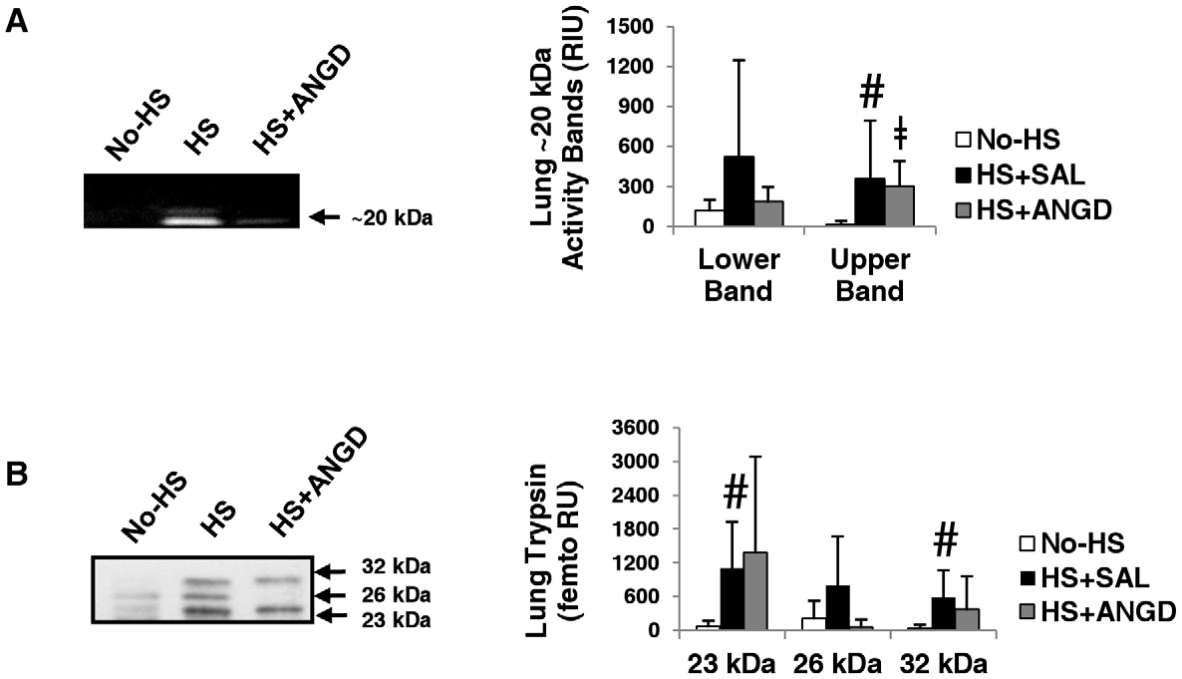
Pancreatic trypsin levels in lung tissue. (**A**) Two activity bands around 20 kDa were detected in lung homogenate by gel zymography. (**B**) Three isoforms of trypsin were detected in lung tissue homogenate using the femto ECL substrate. The 32 kDa band corresponds to the molecular weight of the trypsin precursor, trypsinogen, whereas the 23 and 26 kDa bands probably correspond to active forms of trypsin. N = 5, 6, 6 rats per group for No-HS, HS+SAL, and HS+ANGD, respectively. #p<0.05 vs. No-HS by Mann-Whitney test; ≠p<0.05 vs. No-HS by t-test.

Lung tissue homogenates also had two distinct bands of low molecular weight protease activity observed by gelatin zymography that were inhibited by renaturing in ANGD (Fig. 5A). We confirmed the proteases were at least trypsin-like in specificity by the failure of the bands to appear as strongly after renaturing and developing the gels in buffers containing TLCK. The upper band was significantly elevated after shock. We noted considerable variability in the presence of the lower band in the HS+SAL group, but no significant differences between the groups. Three isoforms (23, 26, and 32 kDa) of pancreatic trypsin were detected by immunoblotting (Fig. 5B) with the 32 kDa band likely corresponding to trypsinogen-3, the zymogen form of trypsin-3 from the pancreas (according to the antibody manufacturer's datasheet). These 23 and 32 kDa isoforms were significantly elevated after hemorrhagic shock in the HS+SAL group. The 26 kDa isoform of pancreatic trypsin was present in all HS+SAL animals but only detected in one lung homogenate from the HS+ANGD group. No isoforms of trypsin were detected in heart or liver tissues by immunoblot (not shown).

### MMP-9 Activity and Concentration

**Figure 6:**
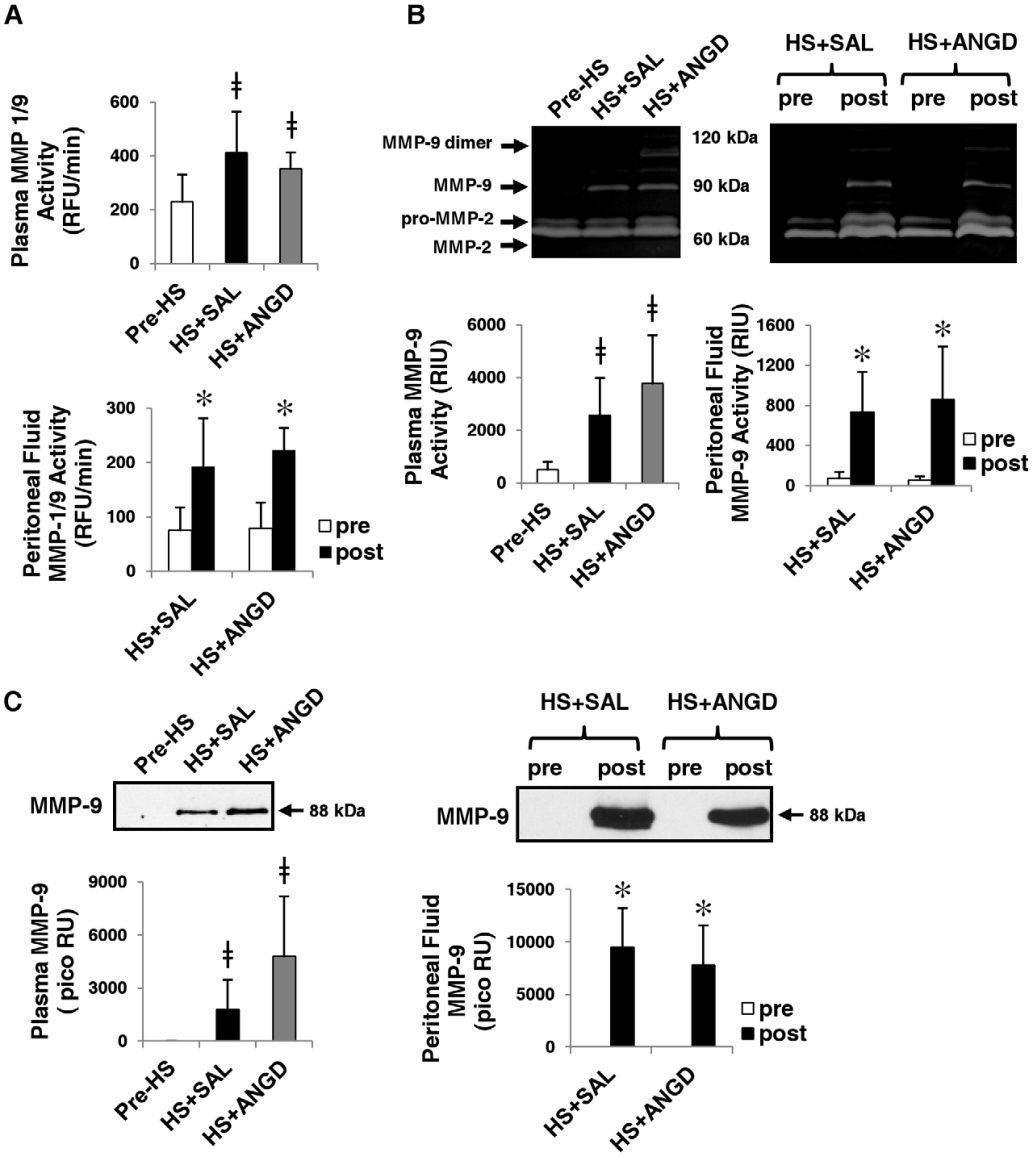
MMP-9 activity and levels in plasma and peritoneal fluid. (**A**) Plate zymography with MMP-1/9 substrate, (**B**) gelatin gel zymography, and (**C**) immunoblots for MMP-9 in plasma (left) and peritoneal fluid (right). MMP-9 activity was elevated by both plate and gelatin gel zymography. There was no change in MMP-2. N = 6, 5, 5 rats per group for plasma and N = 6 rats per group for peritoneal fluid. Immunoblotting quantification is reported in RU with the pico ECL substrates. ≠p<0.05 vs. Pre-HS group by t-test; *p<0.05 by paired t-test comparing pre vs. post HS+SAL or HS+ANGD.

MMP-9 activity was significantly elevated after shock compared to pre-shock levels as detected by plate zymography with the MMP-1/9 specific substrate (Fig. 6A). Gelatin gel zymography also revealed elevated MMP-9 bands in hemorrhagic shock that were undetectable in pre-shock plasma and peritoneal fluid (Fig. 6B). Immunoblot results for total protein confirmed the presence of MMP-9 in post-HS plasma and peritoneal fluid regardless of pre-treatment with ANGD, but little to no MMP-9 before shock (Fig. 6C). On average, MMP-9 dimer activity was increased in the plasma of ANGD treated animals (undetectable in HS+SAL group).

**Figure 7:**
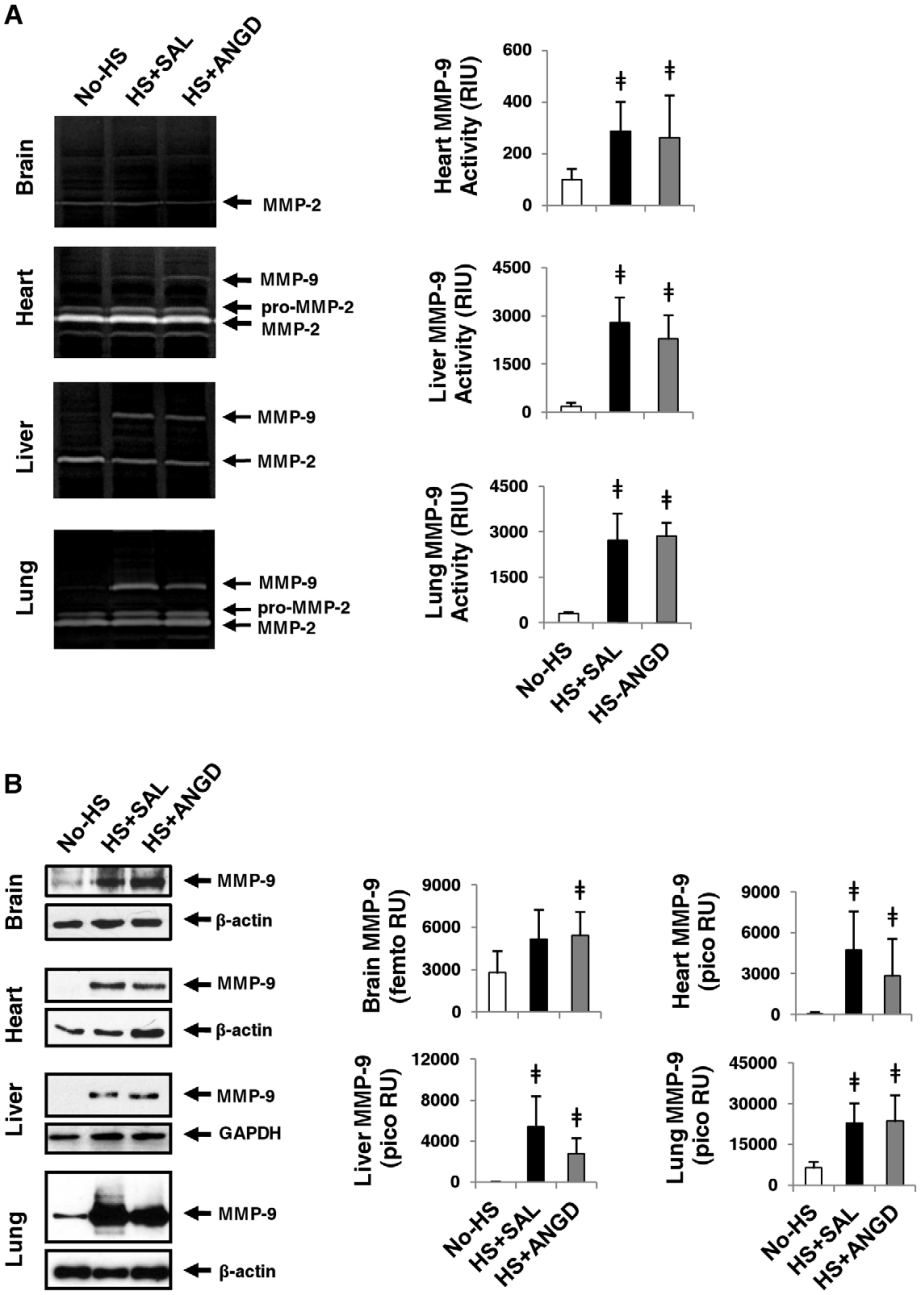
MMP-9 activity and levels in vital organs. (**A**) Gelatin gel zymography revealed increased levels of MMP-9 activity in the heart, liver, and lung (quantifications to the right) after hemorrhagic shock. There was no change in MMP-2. (**B**) Immunoblot detection of MMP-9 was upregulated in brain (bands detected with femto ECL substrate), heart, liver, and lung (bands detectable by pico ECL substrate). N = 5 rats per group, ≠p<0.05 by t-test vs. No-HS.

Lung, liver, and heart homogenates also had elevated MMP-9 activity levels after shock measured by gelatin gel zymography (Fig. 7A). Immunoblot analysis revealed detectable MMP-9 in all organs after shock, although femto chemiluminescent detection of MMP-9 protein levels was necessary to visualize bands in the brain homogenate (Fig. 7B).

Both pro-MMP-2 and MMP-2 were detected by gel zymography in all samples, but there were no differences between the groups after quantification (not shown).

Since MMP-9 can be directly activated by serine proteases, we collected blood every 15 minutes during the first hour of ischemia of HS+SAL animals to determine the order of appearance of MMP-9 and serine proteases in the plasma as measured by gelatin gel zymography and immunoblots. In all animals (N = 3), bands at the molecular weight of active MMP-9 appeared in gelatin gel zymography and immunoblots by 15 minutes, whereas serine protease bands in the zymography or trypsin levels measured in the immunoblot did not distinctively appear at all during the first hour of ischemia, suggesting MMP release and initial activation in HS did not depend on entry of serine proteases into the circulation.

## Discussion

Following hemorrhagic shock, protease activities by trypsin-, chymotrypsin-, elastase-like enzymes, and MMP-9 were significantly elevated in plasma and peritoneal fluid when measured by specific small substrates and gelatin gel zymography. However, a general substrate, casein, detected the elevation of activity in peritoneal fluid but not in plasma. Immunoblotting confirmed the transport of pancreatic trypsin and the presence of increased levels of MMP-9 activity after hemorrhagic shock in the plasma, peritoneal fluid, and peripheral organs.

An active form of trypsin was detected in peritoneal fluid only after shock, indicating a direct penetration of trypsin into the peritoneal fluid from the intestinal lumen or pancreas. This evidence suggests that after hemorrhagic shock not only the intestinal mucosal barrier but also the entire intestinal wall in the rat may become permeable to shock mediators and to digestive enzymes [20]. Increased permeability could explain the presence of inflammatory mediators in the peritoneal fluid after shock [11].

Serine proteases may also be transported by the mesenteric lymph fluid and enter into the circulation via the thoracic duct. The lymph fluid mixes with the blood before entering the capillary bed in the lung, one of the first organs to fail in shock [12], [21]. While the increase in *plasma* trypsin levels after shock as measured by immunoblotting was moderate and non-significant (femto ECL substrate was necessary to visualize the bands by immunoblotting), three isoforms of pancreatic trypsin were detected in the lung homogenate and two of the three were significantly elevated in hemorrhagic shock. Thus, it is possible that pancreatic intestinal proteases transported through the lymphatic fluid become entrapped in lung tissue, which is susceptible to increased vascular permeability and protein uptake [22]. The inability to detect pancreatic trypsin in the liver may indicate that transport of intestinal proteases at this early stage of hemorrhagic shock predominantly is through the lymph fluid and peritoneal fluid rather than via the portal venous blood.

Active MMP-9 was elevated in all samples after hemorrhagic shock, in line with reported events during ischemia-reperfusion in organs, including non-intestinal organs [23]–[26]. *De-novo* transcription of MMP-9 requires several hours and is less likely a major source at the two-hour reperfusion collection in the current study, than release of existing MMP-9. Given that endothelial cells, neutrophils, and monocytes are all sources of MMP-9 [27], [28] and MMP-9 levels were all increased after the ischemia/reperfusion induced by hemorrhagic shock, MMP-9 may be activated by the ischemia associated with hemorrhagic shock rather than only by specific events in the intestine. This hypothesis is supported by our finding that MMP levels are increased in plasma at 15 minutes of ischemia, while pancreatic proteases remain undetectable with current techniques in the plasma through the first hour of ischemia. Transport of the serine proteases may occur predominately during reperfusion, when the majority of injury during shock occurs, rather than during ischemia [29]. Since we did not detect MMP-9 in zymogen form in the plasma during the first hour of ischemia, it is less likely that serine proteases entering upon reperfusion would convert appreciable amounts of zymogen MMP-9 into its active form, though serine proteases may stimulate further MMP release. We have shown previously that MMPs in the ischemic intestinal wall are activated by trypsin entering from the intestinal lumen [6], so it is also possible that *additional* active MMP-9 from the intestine or pancreas enter the circulation during reperfusion.

ANGD in the intestinal lumen reduces protease activity in an ischemic intestine, morphological damage to the villi and systemic inflammation [8]. Other serine protease inhibitors with similar structure give similar protective effects when enterally administered [30]. Our results also show that gross morphological damage to the intestine and neutrophil infiltration in peripheral organs as measured by organ MPO levels are decreased with ANGD treatment. However, at 2 hours after hemorrhagic shock ANGD had little effect on protease activities measured in compartments distant from the intestine (plasma, as well as lung, liver, heart, and brain homogenates). The limited effect may be due in part to the ischemic pancreas acting as a source of plasma proteases that would not be affected by ANGD in the lumen of the intestine. This hypothesis is supported by the presence of a trypsinogen band in the lung and by the fact that the 26 kDa band of trypsin, the only band of trypsin detected in peritoneal fluid, was absent from all but one of the lung homogenates in the HS+ANGD group, while 23 kDa and 32 kDa (trypsinogen) bands were unaffected by intestinal ANGD treatment.

Enterally administered ANGD prevented organ neutrophil infiltration, but did not significantly lower protease activities, suggests that neutrophil infiltration is facilitated by the low flow state in conjunction with shock mediators other than circulating proteases. This hypothesis is supported by the limited efficacy in *acute* shock of ANGD given i.v. as opposed to administration into the intestinal lumen [31]. We hypothesize that there are multiple categories of shock mediators derived from digestive enzymes or their byproducts, which may enter into the intestinal wall and may be affected by enzyme inhibitor treatment in the lumen of the intestine. These mediators may cause distant site injury after transport from the ischemic intestine via the circulation. Besides the digestive enzymes we focused on here, they include: 1) bioactive peptides formed by proteolytic cleavage of extracellular proteins in the intestine [32], 2) free fatty acids formed by digestive lipases acting on intestinal cells and/or dietary fat in the intestinal lumen, which may damage cells directly through a detergent mechanism [5], [33], and 3) damage associated molecular pattern molecules (DAMPs) released from dead cells in the intestine (e.g. after free fatty acid-induced necrosis) [32]. It is possible that these latter mediator types may be involved in neutrophil infiltration into organs distant from the intestine in hemorrhagic shock.

ANGD in the lumen of the intestine also had the surprising effect of increasing plasma trypsin-like activity, as well as gelatinolytic activity in a band at the molecular weight of trypsin. Plasma trypsin protein concentration, however, as determined by immunoblot was no different after HS with saline versus with ANGD, suggesting that the presence of ANGD may have caused the trypsin that was crossing into the blood to become modified to a more active form or activate other trypsin-like enzymes of a similar molecular weight, rather than increasing the actual trypsin present.

We made use of both large and small protease substrates in this study in order to compare their efficacies in detecting protease activity in plasma. Casein is a large globular substrate that can be collectively cleaved by multiple protease classes (serine proteases, MMPs, sulfhydryl, and acid proteases, etc.), making it a useful substrate for detection of general proteolytic activity. Though it is possible that, given a longer digestion time, plasma digested casein may yield an improved fluorescent signal, we observed at 2 hours reperfusion only low levels, barely above those measured by buffer alone. Despite this, we were able to detect differences between pre- and post-HS plasma when PMSF was used to block serine proteases. The remaining activity could be from MMPs such as MMP-9, which is increased after HS (Fig. 6). However, casein was not able to detect the significant increase in overall activity in plasma after shock that we observed using smaller, more specific substrates.

Given that the small peptide substrates were significantly cleaved despite levels of activity of only nanomolar concentrations of trypsin, chymotrypsin, and elastase, whereas casein was not able to detect differences before and after HS, casein may not be the optimal choice for detection of protease activity in shock plasma. Our findings support the evidence that plasma protease inhibitors such as α_2_-macroglobulin, α_1_-antitrypsin, etc., only prevent pancreatic proteases from hydrolyzing larger globular proteins such as casein, but do not inhibit their activity on smaller substrates [17]. Instead, either the actual protein of interest should be used as a substrate, or in lieu of that, small peptide substrates alone or in combination with the hypothesized substrate may be better able to reveal the proteolytic potential in a given plasma sample.

The intestine is a key organ in the etiology of shock and if the release of mediators from the intestine is limited by ligation of the major mesenteric lymph vessels, both lung injury and mortality are reduced [12]. Several intestine-derived shock mediators have been proposed, but because of the presence of plasma protease inhibitors, digestive enzymes have mostly been discounted as potential shock mediators distant from the intestine itself. However, active proteases are capable of degrading surface receptors such as the insulin receptor or activating receptors such as the protease-activated receptors [13], [34]. Furthermore, MMP-9 cleaves vascular endothelial cadherin, which is important to maintain endothelial permeability, and is disrupted during shock [35], [36]. Cytokines pro-IL-1β and pro-TNFα are processed to their active form by MMP-9 and are involved in vascular inflammation during shock [37]–[40]. Though early neutrophil infiltration into organs distant from the intestine may not be affected by plasma, peritoneal, and organ protease activities, it is likely that later events in shock, such as insulin resistance, may be linked to the action of proteases. Thus circulating proteases may represent a distinct damage mechanism. Organ failure in shock may be the result of the synergy of multiple damage mechanisms.

We have shown here that both serine and MMP proteolytic activity is increased early (2 hr ischemia/2 hr reperfusion) after shock in plasma, peritoneal fluid, and organs distant from the intestine, especially the lung, which is particularly vulnerable to damage during shock. The increase in protease presence and activities may contribute to the lethal progression of shock and may be a target for therapeutics.
